# Voided volume is important to prevent stone recurrences

**DOI:** 10.1002/bco2.273

**Published:** 2023-09-21

**Authors:** Felipe Pauchard, Frederic Panthier, Olivier Traxer

**Affiliations:** ^1^ Urology Department Hospital Naval Almirante Nef Viña del Mar Chile; ^2^ Progressive Endourological Association for Research and Leading Solutions (PEARLS) Paris France; ^3^ GRC n820, Groupe de Recherche Clinique sur la Lithiase Urinaire, Hôpital Tenon, Sorbonne Université, bService d'Urologie, Assistance‐ Publique Hôpitaux de Paris, Hôpital Tenon, Sorbonne Université Paris France; ^4^ PIMM, UMR 8006 CNRS‐Arts et Métiers ParisTech Paris France

We read with interest the study by Kevin Shee and colleagues,^1^ reporting how urinary volume does not impact on stone recurrence, based on a local stone registry. This study firstly challenges the urinary volume paradigm in stone disease. Indeed, increasing fluid intake to prevent urolithiasis is one of the most established and effective recommendations in the management of stone former patients.^2^ Paradigms must be challenged to keep moving forward, and science is full of examples. Nicolaus Copernicus and his heliocentric theory is probably one of the most knowns paradigm shifts. Anyhow, caution should be taken with the evidence that support such propositions. In this specific case, the authors included patients that had a 24 h urine collection and at least one stone event and divided them in two groups according to the number of stone events (1 vs. ≥2). No data on the number of kidney stones at time of 24 h urine collection, residual fragments after surgery and time of follow‐up are provided. This introduces an immortal time bias, since the two groups were divided based on their baseline characteristics (i.e. number of stone events). We ignore if the cohort of patients included in this study already had a throughout counselling about kidney stone disease, since both groups had a comparable mean daily voided volume of more than 2 L, which is already very well in agreement with current recommendations.^2^ When selection bias occurs in a clinical trial, the two study groups may differ considerably regarding known and unknown covariates at baseline, before treatment begins. As a result, any differences between the two groups at the end of the trial could be caused by either the intervention being evaluated or the baseline differences.^3^

Stone formation is a multifactorial process,^4^ resulting in supersaturation of the urine. Recurrent stone formers met this prerequisite due to low urine volume, higher urine calcium and oxalate concentrations.^5^ There are randomised prospective studies that have demonstrated that increasing voided volume decreases stone events roughly by half over 5 years.^6^ It has been stated that for each additional 200 mL of fluids consumed per day, the risk of stone formation is reduced by 13%.^7^ It must be taken into account also what kind of beverage are the patients drinking and its timing.^8^, ^9^ Nevertheless, fluid intake is not the only variable that can increase stone formation, as mentioned before, but is the only one assessed in this study, despite having a comparatively adequate diuresis in both groups. Also, cystine patients were excluded from analysis, but this aspect is mentioned only in the method section. Therefore, the voided volume impact on this patients is not challenged by this work, which has to be highlighted as we know these type of stones require an even higher daily voided volume (>3 L/24 h) to diminish recurrences.^10^ For all other stone types, except calcium oxalate monohydrate stones, the number of included stones was less than 30 and renders statistical analysis underpowered.

We encourage to challenge paradigms to keep moving forward, but we think that good quality data are lacking nowadays to challenge high volume diuresis for stone prevention. We also agree that prospective studies are needed to confirm previous studies on high volume diuresis and urine density. In the meantime, stone formers should still be encouraged to have a diuresis of more than 2 L per day.

## DISCLOSURE

Prof. Olivier Traxer is a consultant for Coloplast, Rocamed, Olympus, EMS, Boston Scientific and IPG.
